# Structure Characterization, In Vitro Antioxidant and Anti-Tumor Activity of Sulfated Polysaccharide from *Siraitia grosvenorii*

**DOI:** 10.3390/foods12112133

**Published:** 2023-05-25

**Authors:** Pin Gong, Mengrao Wang, Yuxi Guo, Hui Long, Zhineng Wang, Dandan Cui, Wenbo Yao, Wenjuan Yang, Fuxin Chen, Jianwu Xie

**Affiliations:** 1 School of Food and Biological Engineering, Shaanxi University of Science and Technology, Xi’an 710021, China; gongpin@sust.edu.cn (P.G.); 13488311504@163.com (M.W.); guoyuxi416@163.com (Y.G.); longhui980525@163.com (H.L.); wangzhineng0102@163.com (Z.W.); cuidandan@163.com (D.C.); yaowenbo@sust.edu.cn (W.Y.); yangwenjuan@sust.edu.cn (W.Y.); 2 School of Chemistry and Chemical Engineering, Xi’an University of Science and Technology, Xi’an 710054, China; chenfuxin1981@163.com

**Keywords:** *Siraitia grosvenorii* polysaccharides, sulfated modifications, anti-tumor

## Abstract

From *Siraitia grosvenorii*, a natural polysaccharide named SGP-1 was discovered, and its purity was determined to be 96.83%. Its structure is a glucan with 4-, 6- and 4,6-linked glucose units. In this paper, the sulfated derivative S-SGP of SGP-1 was prepared by the chlorosulfonic acid method. The sulfated derivatives were analyzed by Fourier transform infrared spectroscopy (FT-IR), gel permeation chromatography (GPC), and scanning electron microscopy (SEM). The degree of substitution (DS) of the polysaccharide is 0.62, and the weight average molecular weight (Mw) is 1.34 × 10^4^ Da. While retaining the morphological characteristics of polysaccharides, S-SGP appeared a large number of spherical structures and strong intermolecular forces. The in vitro activity study of S-SGP showed that the sulfated derivatives had the ability to scavenge DPPH radicals, hydroxyl radicals and superoxide anions, and the scavenging power tended to increase with the increase in polysaccharide concentration. It can inhibit the growth of human hepatoma cells (HepG2), human breast cancer cells (MDA-MB-231) and human non-small cell lung cancer cells (A549) in vitro. In addition, the treatment of A549 cells with sulfuric acid derivatives can decrease the mitochondrial membrane potential, induce apoptosis, and alter the expression of apoptosis-related mRNA and protein.

## 1. Introduction

*Siraitia grosvenorii* (SG) is a dried fruit of the *Cucurbitaceae* plant *Siraitia grosvenorii* (*Swingle*) *C. Jeffrey*. SG is the first batch of Chinese herbal medicines approved by the state for medicinal and edible purposes [1]. Pharmacological studies have shown that SG has physiological functions such as antibacterial, anti-tumor, regulating the immune system, and lowering blood sugar [2,3,4]. *Siraitia grosvenorii* polysaccharide (SGP) is a polymeric carbohydrate macromolecule extracted from SG [3]. Lin et al. [5] have shown that SGP can not only improve lipid metabolism disorders, but also reduce blood sugar levels, and its hypoglycemic mechanism may be related to improving blood lipid metabolism and restoring blood lipid levels. The ultrasonic-assisted extraction technique was used by Chen et al. to extract SGP, and they discovered that it effectively scavenges OH, DPPH, and Fe3+ [6]. Gong et al. [3] isolated a new polysaccharide from SG that could reduce the inflammatory response in diabetic nephropathy (DN) mice through the TLR4/NF-*κ*B pathway. It is evident that SGP exhibits promising biological activity due to its ability to regulate lipid metabolism and possess antioxidant functions. Therefore, it holds the potential to improve diseases characterized by lipid metabolism disorders, such as obesity and non-alcoholic fatty liver disease. In the structural study of SGP, it was found that the main chain of SGP is a polysaccharide consisting of both *α*-glycosidic and *β*-glycosidic linkages. The side chains are formed by *β*-D-Gal*p* and *α*-D-Glc*p* units attached at the C_6_ position of Glc*p* and Man*p*, respectively [7]. Additionally, in this study, it was observed that SGP exhibits significant hypoglycemic activity. However, there is a lack of research on sulfated polysaccharides derived from SGP. In order to fill this research gap, we performed sulfation of SGP to further investigate its properties and provide a basis for the development of potential applications.

Chemical modification of polysaccharides is the main strategy for studying the structure–activity relationship of polysaccharides [8]. Acetylation and sulfation are common modification methods [9] in natural polysaccharides. However, there are few studies on the structural modification of SGP and its influence on biological activities. Sulfated polysaccharides refer to polysaccharides with sulfate groups substituted on the sugar chains. This includes naturally occurring sulfated polysaccharides extracted from algae [10,11] as well as chemically sulfated polysaccharides obtained through functional group substitution [12,13,14]. The introduction of sulfate groups to polysaccharides alters their biological activity. This is due to changes in spatial hindrance, electrostatic repulsion, chain flexibility, and water solubility caused by the presence of sulfate groups [15]. Current research has unveiled the diverse biological activities of sulfated polysaccharides, including anti-tumor effects [16,17], immune regulation [18,19], antiviral activity [20] and antioxidant properties [21,22]. Therefore, structural modification of polysaccharides can be advantageous in enhancing their biological activity [23].

To further explore the structure–activities of SGP, a sulfated SGP has been studied by an in vitro anti-oxidant assay and anti-tumor experiment. Through comparative analysis with SGP, our study aims to elucidate the impact of side-chain group modifications on biological activities. This investigation may offer valuable theoretical insights for the future development and utilization of SG plant resources.

## 2. Materials and Methods

### 2.1. Materials and Chemicals

*Siraitia grosvenorii* specimens were collected in Guilin, Guangxi Province, China. Xi’an Medical College (Xi’an, Shaanxi) generously donated human breast cancer cells (MDA-MB-231), human hepatoma cells (HepG2) and human non-small cell lung cancer cells (A549). Chlorosulfonic acid, concentrated hydrochloric acid, trifluoroacetic acid, sodium hydroxide and pyridine were obtained from Sinopharm Chemical Reagent Company; Annexin V-FITC apoptosis kit, BCA protein concentration assay kit and reactive oxygen species (ROS) detection kit were obtained from Beyotime Biotechnology (Shanghai, China); the mitochondrial membrane potential assay (JC-1) kit, 2× SYBR Green and qPCR Master Mix (Low ROX) were purchased from Servicebio (Wuhan, China); rabbit anti-Cyt-C monoclonal antibody, rabbit anti-Bcl-2 monoclonal antibody, rabbit anti-Bax monoclonal antibody, rabbit anti-Caspase-3 monoclonal antibody and *β*-action antibody were obtained from Cell Signaling Technology (CST, Boston, MA, USA).

### 2.2. Isolation and Purification of the Siraitia Grosvenorii Polysaccharide

SGP-1 was isolated from *Siraitia grosvenorii* using the methodology outlined in our previous publication [24]. Briefly, the proteins were eliminated using the Chloroform and n-butanol (4:1, *v*/*v*) followed by purification using a DEAE cellulose column. The resulting eluate was concentrated, dialyzed with distilled water for 24 h, and subjected to additional purification using Se-phadex G-200 (Shanghai Huicheng Biotechnology Co., Shanghai, China). This process resulted in the isolation of a white, sparsely purified polysaccharide from *Siraitia grosvenorii*, designated as SGP-1.

### 2.3. Characterization of Siraitia Grosvenorii Polysaccharide (SGP-1)

#### 2.3.1. Analysis of the Polysaccharide Content

The polysaccharides were determined by reference to the method previously reported by our group [1,2,25]. The total saccharide content was measured by the phenol-sulfuric-acid method. A standard curve for polysaccharides was constructed using glucose as the standard compound. The content of polysaccharide was determined using the phenol sulfuric acid method and the absorbance (A) at 490 nm was recorded using a multifunctional enzyme-linked immunosorbent assay analyzer provided by Thermo Fisher (Wilmington, NC, USA). To determine the polysaccharide content, 1 mL of the SGP solution was accurately measured, followed by the addition of a phenol sulfuric acid reagent. Distilled water was a blank sample. Afterward, the mixture was subjected to heating in a boiling water bath for 10 min, followed by measurement of the absorbance at 490 nm. Repeated three times, and the total saccharide content was calculated accordingly.

#### 2.3.2. Determination of Molecular Weight (Mw) and Monosaccharide Compositions

Using a gel filtration chromatography system from Malvern Panalytical (Shanghai, China) to determine the molecular weight size. The SGP-1 was dissolved in an aqueous solution of 0.1 M NaNO_3_ (SGP-1: 1 mg/mL). The solution of SGP-1 was passed through a 0.45 μm microporous membrane, injected into the system, and the retention time was recorded. Calculate the molecular weight of the polysaccharide. The chromatography system adopts a gel chromatography differential–multi-angle laser light scattering system [26,27]. According to the properties of polysaccharides, a gel size exclusion chromatography column with a corresponding molecular weight range is selected. The column temperature is set to 45 °C, 100 μL is injected, and the mobile phase is 0.1 M NaNO_3_, 0.4 mL/min isocratic elution for 100 min [28].

A mixed standard solution was prepared by dissolving individual monosaccharide standard products, including galacturonic acid (GalAc), glucuronic acid (GlcAc), fucose (Fuc), galactose (Gal), xylose (Xyl), mannose (Man), rhamnose (Rha), glucose (Glc), arabinose (Ara) and ribose (Rib), in an appropriate solvent. To determine the monosaccharide composition of SGP-1, 10 mg of SGP-1 was weighed and mixed with 10 mL of trifluoroacetic acid. The mixture was sealed and hydrolyzed at 120 °C for 3 h. After nitrogen blow-drying, we added 10 mL of water to the residue, followed by thorough mixing. Subsequently, 100 μL of the solution was taken and diluted with 900 μL of ultrapure water. The resulting mixture was centrifuged (12,000 rpm, 5 min), and a precise volume of 5 μL of the supernatant was carefully collected for subsequent monosaccharide composition analysis. A DionexCarbopacTMPA20 (3 × 150 mm) chromatographic column was used with the following mobile phases: A: H_2_O; B: NaOH (15 mM); C: NaOH (15 mM) and NaOAc (100 mM); the temperature of the column was kept constant at 30 ℃; the flow rate was set to 0.3 mL/min [29].

#### 2.3.3. Ultraviolet (UV) and Fourier Transform Infrared (FT-IR) Spectra Analysis

In this experiment, we were using a UV spectrophotometer from Shanghai Pulei Instrument Co., Ltd. (Shanghai, China) to measure UV, and a FT-IR spectrometer from Thermo Fisher Scientific (Wilmington, NC, USA) to measure UV. A precise amount of SGP was carefully weighed and dissolved in distilled water to obtain a solution (0.5 mg/mL). Subsequently, UV scanning was conducted over the wavelength range of 200–400 nm [30].

The FT-IR spectrum of SGP-1 was obtained using the KBr pellet method, with the wavelength range recorded from 4000 to 400 cm^−1^ [29].

#### 2.3.4. Morphology Analysis

High-resolution field emission scanning electron microscopy (SEM) was employed for microstructural observations, provided by FEI Company (Eindhoven, The Netherlands). Take a suitable size of conductive adhesive and stick it on the scanning electron microscope (SEM) sample stage, dip an appropriate amount of SGP-1 powder on the conductive adhesive, gently blow off the unabsorbed polysaccharide with an ear-washing ball, spray with gold for 15 s, under different magnifications. The microscopic morphology of SGP-1 was observed by SEM [31].

### 2.4. Sulfated Modification of SGP-1

Currently, there are several methods available for the preparation of sulfated polysaccharides, including the chlorosulfonic acid-pyridine method [32], concentrated sulfuric acid method [33] and sulfur trioxide-pyridine method [34]. The chlorosulfonic acid-pyridine method is widely utilized among these methods. It offers advantages such as high yield and convenient operation. In the application of this method, the ratio of chlorosulfonic acid to pyridine, as well as the reaction temperature and time, are important influencing factors [35]. The concentrated sulfuric acid method has the disadvantages of polysaccharide degradation and carbonization, while the sulfur trioxide-pyridine method involves a complex preparation process. Therefore, the chlorosulfonic acid-pyridine method reported by Wang et al. was employed for the preparation of sulfated polysaccharides [36].

Weighing 500 mg of SGP-1, it was added to a solution containing 30 mL of anhydrous dimethylformamide (DMF), followed by continuous stirring for 30 min until dissolution. The obtained solution was transferred to a three-necked flask containing the sulfation reagent for reaction (60 °C, 2 h). Then, cooling to room temperature, a small amount of distilled water was added to facilitate phase separation and settling. The solution was then adjusted to the pH of 7.0 using NaOH (4 mol/L). Centrifuge the solution and collect the supernatant (3500 rpm, 10 min). To precipitate the desired product, added ethanol was added (3:1, *v*/*v*) and allowed to stand for 24 h. After another centrifugation step of 10 min, the resulting precipitate was collected. The precipitate was dissolved in distilled water, followed by dialysis for 48 h using ultrapure water, and finally subjected to freeze-drying, resulting in the S-SGP product.

### 2.5. Characterization of Sulfated Polysaccharide (S-SGP)

#### 2.5.1. Degree of Substitution (DS)

The degree of DS of a polysaccharide represents the average number of sulfate residues substituted on each monosaccharide residue. Typically, barium chloride-gelatin turbidimetry was used to determine DS [37]. The DS in the SGP-1 is calculated using the following formula [38].

(1) $$\mathrm{DS}=\frac{1.62\;\times \;S\%}{32\;-\;1.02\;\times \;S\%}$$

In the formula (1): *S*% indicates the sulfur content.

#### 2.5.2. Analysis of the Polysaccharide Content, Mw, FT-IR and Morphology Analysis

Refer to Section 2.3 for relevant measurement methods.

### 2.6. In Vitro Antioxidant Assay

#### 2.6.1. OH Scavenging Activity

Polysaccharide solution (2 mL) with different concentrations (0.125, 0.25, 0.5, 1, 2, 4 mg/mL), FeSO_4_ solution (2 mL), H_2_O_2_ (2 mL) and salicylic acid-ethanol solution (2 mL) was added to the 10 mL colorimetric tube in turn, after mixing, let stand for 1 h at 25 °C, with ascorbic acid as positive control and water as blank control, measure the absorbance of each tube at 510 nm [39].

#### 2.6.2. DPPH Scavenging Activity

In a 10 mL colorimetric tube, polysaccharide solution (2 mL) with different concentrations (0.125, 0.25, 0.5, 1, 2 and 4 mg/mL) and DPPH solution (2 mL) were successively added. The mixture was then kept in a light-protected environment at room temperature for a duration of 30 min. A blank control containing water and a positive control using ascorbic acid were included for comparison. The absorbance of the mixture was then evaluated at 517 nm to assess its antioxidant activity [40].

#### 2.6.3. O_2_^−^∙ Scavenging Activity

In test tubes with stoppers, polysaccharide solutions (1 mL) with different concentrations (0.125, 0.25, 0.5, 1, 2, and 4 mg/mL) were added. Following that, Tris-HCl (3 mL, pH 8.2) buffer solution was introduced, and the solution was incubated in a water bath (30 °C, 20 min). Subsequently, the tubes were placed at room temperature, and pyrogallic acid solution (3 mL, 5 mmol/L) was added. The contents were thoroughly mixed for 3 min, and the reaction was halted by adding concentrated hydrochloric acid (1 mL). A blank control using water and positive control with ascorbic acid was included for comparison. The absorbance was recorded at 320 nm to assess the antioxidant activity [41].

### 2.7. Anti-Cancer and Apoptosis Assay

#### 2.7.1. Cell Lines and Cultures

The human breast cancer cell line (MDA-MB-231), human hepatoma cell line (HepG2) and human non-small cell lung cancer cell line (A549) were provided by the nutrition and health cell platform of Shaanxi University of Science and Technology School of Food and Biological Engineering and maintained with Dulbecco’s Modified Eagle’s Medium (DMEM, a commonly used cell culture medium that is widely utilized for in vitro cultivation of various mammalian cells) high glucose medium, containing 1% Penicillin streptomycin and 10% fetal bovine serum. Incubate in a humid incubator containing 5% CO_2_ at 37 °C.

#### 2.7.2. Growth Inhibition Assay

The inhibitory effect of SGP-1 and S-SGP on HepG2 cells, A549 cells and MDA-MB-231 cells in vitro were studied by Cell Counting Kit-8 (CCK-8, a commonly used reagent kit for assessing cell viability) method. They were seeded in a 96-well plate at about 5000 cells/well, 100 μL per well, and pre-cultured in an incubator for 24 h. Then, different concentrations (0, 12.5, 25, 50, 100, 200, 400 μg/mL) of S-SGP solution and 200 μg/mL 5-Fluorouracil (5-FU, an anticancer drug widely used for the treatment of various malignant tumors) solution was added, respectively. After 48 h of incubation at 37 °C, we added CCK-8 solution (10 μL) to each well and continued to incubate for 4 h in the incubator. After removing the microplate from the shaker, the contents were gently mixed. Then, the absorbance of the 96-well plate was measured at 450 nm using a microplate reader. To evaluate the results, the absorbance of the polysaccharide-treated group was compared with that of the solvent control group. The positive control in this experiment was 5-FU.

#### 2.7.3. Apoptosis of A549 Cells

Cells (2 × 10^5^ cells/mL) were incubated in 6-well plates with different concentrations of S-SGP solutions (0, 100, 200, 400 μg/mL), and 5-FU (200 μg/mL) was used as a positive control. After 48 h of incubation, cells and culture medium were collected, centrifuged (1000 rpm, 5 min) and the supernatant was discarded; 195 μL of Fluorescein Isothiocyanate-labeled Annexin V (Annexin V-FIT, a commonly used fluorescent probe for detecting cellular apoptosis) conjugate was added to gently resuspend the cells, then Annexin V-FITC (5 μL) was added, gently mixed and then propidium iodide (PI) staining solution (10 μL) was added to mix again. Incubate with aluminum foil for 15 min at 25 °C in the dark, resuspend the cells 3 times during this period, place in an ice bath, and analyze by flow cytometry (BD) immediately.

Take 1.5 mL of A549 cell suspension in the logarithmic growth phase and culture it in a 12-well plate for 24 h. When the cells grow to about 70%, S-SGP solutions with different concentrations (0, 100, 200 and 400 μg/mL) are added and 5-FU (200 μg/mL) as a positive control, and cultured for 48 h. We remove the supernatant, fix the cells with 4% paraformaldehyde for 20 min, then remove the paraformaldehyde solution by suction, wash with PBS, add 0.5 mL of DAPI staining solution to ensure that the cells can be covered, and incubate at 37 °C in the dark for 30 min. After incubation, we added PBS and washed it twice. Under the excitation of ultraviolet light, the morphology of the nucleus was observed and recorded with a fluorescent inverted microscope.

#### 2.7.4. Apoptosis Mechanism of A549 Cells

A549 cells were seeded at a density of 5 × 10^5^ cells/mL in 6-well plates. The plates were then incubated at 37 °C for approximately 24 h. Next, 1 mL of S-SGP solutions with different concentrations (0, 100, 200 and 400 μg/mL) were added to the cells, and co-culturing was continued for 48 h. Incubate with 1 mL of 10 μg/mL Rosup for 15 min as a positive control, discard the culture medium, add 1 mL of 10 μM/L 2′,7′-dichlorodihydrofluorescein diacetate (DCFH-DA, a fluorescent probe commonly used for detecting the generation of intracellular ROS) to each well, and incubate for 20 min in an incubator. After the incubation period, the cells were subjected to three washes with a serum-free medium. Subsequently, fluorescence inverted microscopy was employed to observe the cells, and Image J software (v1.8.0) was utilized to analyze the fluorescence intensity.

Using 200 μg/mL 5-FU as a positive control, cells were harvested after 48 h of incubation with different concentrations of S-SGP (0, 100, 200, 400 μg/mL). Total RNA was extracted using RNeasy Mini-Kit according to the manufacturer’s instructions, and the concentration and purity of the extracted RNA were measured with an ultra-micro UV spectrophotometer. A ratio of *OD_260_/OD_280_* between 1.8 and 2.0 indicated the acceptable purity of the RNA. Total RNA was reverse transcribed with reverse transcriptase according to the manufacturer’s protocol (MightyScript First Strand cDNA Synthesis Master Mix Kit, Sangon, China). The full sequences of *Cyt-C*, *Caspase 9*, *Bcl-2*, *Bax* and *Caspase 3* genes were all obtained from Genebank. Premier 5.0 software was used to design the primers of the six genes synthesized by Sangon Bioengineering Company. The primer sequences are shown in the Table S1. The qRT-PCR was performed using a 2× SYBR Green qPCR Master Mix (Low ROX) reaction system and the reaction program settings pre-denaturation were as follows: 95 °C, 30 s, 40 cycles of denaturation; 95 °C, 15 s, annealing/extension: 60 °C, 30 s.

S-SGP was co-cultured with A549 cells for 48 h. After the cells were washed twice with cold PBS, we added lysis buffer (1 mL). The mixture was then incubated on ice for 30 min, and the supernatant was collected by centrifugation. A BCA kit was used to measure the concentration of protein. Equal amounts of protein were separated using 10% SDS-PAGE, followed by transfer into a PVDF membrane. The membrane was then combined with specific antibodies. Finally, protein detection was performed by ECL reagent.

## 3. Results and Discussion

### 3.1. Characterization of SGP-1

The polysaccharide content of SGP-1 was determined as 96.83%. There are no obvious absorption peaks at 280 nm and 260 nm in UV scanning, which indicates that SGP-1 has been purified without macromolecules such as nucleic acids and proteins. The calculated molecular weight is 3.3 × 10^4^ Da and Mw/Mn = 2.62. The molecular weight of SGP polysaccharide in another study on *Siraitia grosvenorii* polysaccharides was reported as 1.93 × 10^4^ Da [6]; this difference in molecular weight could be attributed to variations in the extraction and purification methods employed in the respective studies.

**Figure 1:**
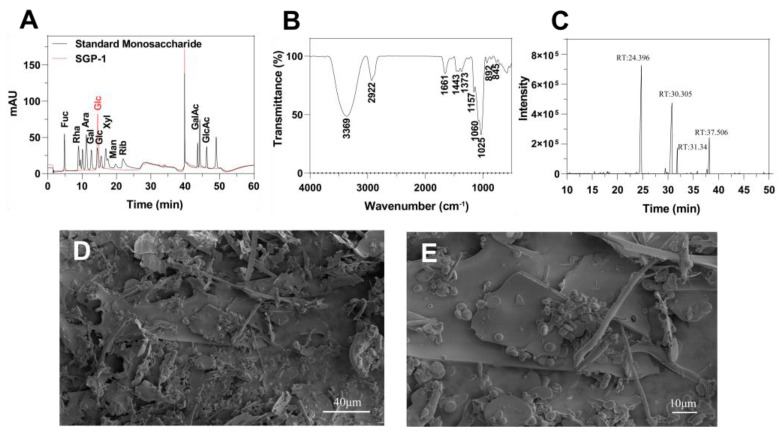
Structural characterization of SGP-1. (**A**) Liquid chromatograms of standard monosaccharides and SGP-1; (**B**) FTIR spectrum of SGP-1; (**C**) GC-MS chromatogram of polysaccharide methylated sugar alcohol acetyl ester (PMAA); (**D**) SEM images of SGP-1 (5000-fold); (**E**) SEM images of SGP-1 (5000-fold).

Figure 1A is a liquid chromatogram of each standard monosaccharide and SGP-1. The chromatogram of SGP-1 shows only one glucose signal, indicating that SGP-1 is glucan.

The FT-IR spectrum of SGP-1, depicted in Figure 1B, exhibits a peak at 3369 cm^−1^ attributed to O-H vibration and a weaker peak at 2922 cm^−1^ associated with C-H vibration. The presence of a peak at 1661 cm^−1^ indicates the stretching vibration of the C=O bond [42]. There are three absorption peaks between 1000 and 1200 cm^−1^, indicating that SGP-1 is pyranose, the three stretching vibration peaks at 1157, 1060 and 1025 cm^−1^ also indicate that it contains dextran, and the absorption band at 1060 cm^−1^ indicates that the glucosyl residue is a glycosidic bond in *β* configuration [43]. The peaks at 892 cm^−1^ and 845 cm^−1^ indicate that both *α*- and *β*-glycosidic bonds exist in SGP-1. In the study on cuaurbit polysaccharide, absorption bands were observed near 892 cm^−1^ and 845 cm^−1^ in the FT-IR spectrum, indicating the presence of *α*- and *β*-glycosidic bonds [44]. Similarly, in another study on SGP, the presence of *β*-glycosidic bonds was also demonstrated [7].

Analysis of polysaccharide methylated sugar alcohol acetyl esters by GC-MS [45], as shown in Figure 1C; four peaks appeared on the chromatogram, identified as 1,5-Di-O-acetyl-1-deuterio-2,3,4,6-tetra-O-methyl-D-glucitol, 1,4,5-Tri-O-acetyl-1-deuterio-2,3,6-tri-O-methyl-D-glucitol, 1,5,6-Tri-O-acetyl-1-deuterio-2,3,4-tri-O-methyl-D-glucitol and 1,4,5,6-Tetra-O-acetyl-1-deuterio-2,3-di-O-methyl-D-glucitol, the molar ratio was 0.486:0.359:0.06:0.095 (Table 1), so they were identified as Glc*p*-(1→, →4)-Glc*p*-(1→, →6-Glc*p*-(1→ and →4,6)-Glc*p*-(1→ [46].

A scanning electron microscope can visually observe the morphology and aggregation state of SGP. Figure 1D is the SEM image of SGP-1 at 2000× magnification, and Figure 1E is the SEM image at 5000× magnification. It can be seen from the figure that SGP-1 has sheet-like, rod-like and spherical structures, which are stacked on each other. It shows that SGP-1 has the main structural features of polysaccharides, with large intermolecular forces and diverse structures.

### 3.2. Characterization of S-SGP

The polysaccharide content of S-SGP is calculated to be 87.67%. According to the calculation formula for the degree of substitution, the DS of S-SGP is 0.62, which indicates that the hydroxyl group in the polysaccharide has been successfully replaced by the sulfate group. The *Mw* of S-SGP is 1.34 × 10^4^ Da, and the *Mw*/*Mn* value is 1.16. The reduced molecular weight compared to SGP-1 may be due to the degradation of the polysaccharide by the strongly acidic reaction environment of the chlorosulfate pyridine method. Cheng et al. also reported a similar phenomenon in their study, where the molecular weight of the polysaccharide decreased during the sulfation process [47]. This decrease in molecular weight could be attributed to the acidic conditions during sulfation, which may have caused the degradation of SGP-1 [48].

**Figure 2:**
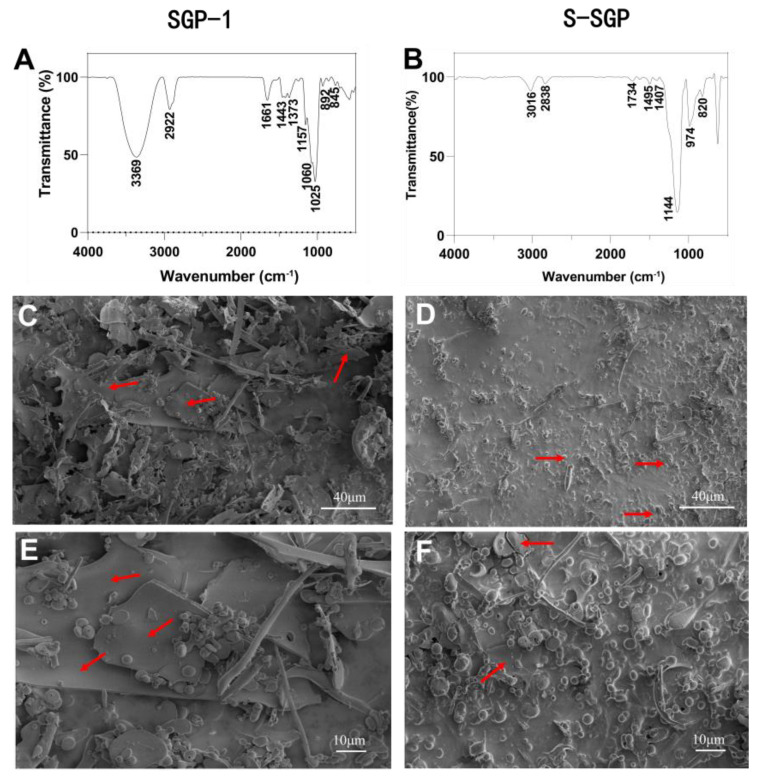
(**A**) FT-IR spectrum of SGP-1; (**B**) FT-IR spectrum of S-SGP; (**C**) SEM images of SGP-1 (2000×); (**D**) SEM images of S-SGP (2000×); (**E**) SEM images of SGP-1 (5000×); (**F**) SEM images of S-SGP (5000×). Following sulfation modification, there is a clear reduction of the presence of lamellar structures within the polysaccharide matrix, accompanied by a prominent increase in the formation of aggregated granular structures.

Figure 2B is the FT-IR chromatogram of S-SGP and the results show that S-SGP has three new absorption peaks at 1260 cm^−1^, 588 cm^−1^ and 820 cm^−1^, which are caused by C-O-S, S-O and O-S-O caused by vibration [49]. It was demonstrated that the hydroxyl group had been converted to the -OSO_3_ group [50]. Compared with SGP-1, the S-SGP sample increased the asymmetric S=O stretching vibration peak of -O-SO_3_ at 1144 cm^−1^ and the C-O-SO_3_ peak at 820 cm^−1^. The stretching vibration peaks of symmetric C-O-S [51] and 624 cm^−1^ are the absorption peaks of sulfate bonds, while the -OH stretching vibration peaks at 3500–3000 cm^−1^ are reduced, indicating that the -OH on the sugar chain has been replaced by sulfate groups.

Figure 2D shows the microscopic features of S-SGP at a magnification of 2000 fold observed by SEM, and Figure 2F shows the microstructure image at a magnification of 5000 fold. It can be seen from the figure that S-SGP has a large number of spherical structures and the morphology is more dispersed, which is consistent with the research findings reported by Huang et al. [52], indicating that sulfation modification changes the microscopic morphology of polysaccharides. Interestingly, in a study on sulfated polysaccharides from Cyclocarya paliurus, it was observed through SEM that the sulfated polysaccharides exhibited a rougher surface [53]. However, in our study, we did not observe evident plate-like structures after sulfation modification. This may be attributed to the spatial hindrance and electrostatic forces caused by the introduction of sulfate groups, which can influence the overall structure. Therefore, the degree of sulfation (DS) is a crucial factor, as revealed by a comparison with the literature. Higher DS values (indicating a greater number of sulfate groups introduced) result in rougher plate-like structures in the polysaccharide [53]. However, the DS of S-SGP in our study is 0.62, higher than the values of 0.42 and 0.12 reported in ref. [53] (where SEM displayed plate-like structures with rougher surfaces). Instead, our polysaccharide exhibited an aggregated particle-like state. Additionally, Huang et al. [52] reported a sulfation degree of 0.52 and also observed a similar particle-like state as ours. Therefore, the changes in the microstructure of the polysaccharide are related to the extent of sulfate group substitution.

### 3.3. In Vitro Antioxidant Activity

In order to evaluate the biological activity of sulfated polysaccharides more comprehensively, in the antioxidant test, the ability of polysaccharides to scavenge DPPH∙ radicals, hydroxyl free radicals and superoxide anions was integrated. Using SGP-1 and S-SGP as materials, the antioxidant properties of polysaccharides before and after sulfated modification were evaluated.

**Figure 3:**
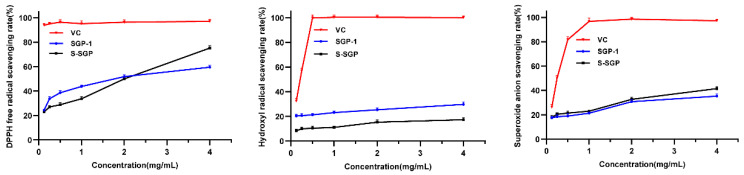
Comparison of antioxidant activity of polysaccharides before and after sulfation modification.

Figure 3 demonstrates the scavenging ability of SGP-1 and S-SGP towards DPPH·. At a concentration of 1 mg/mL, the polysaccharide exhibited an enhanced ability to scavenge DPPH. At this time, the DPPH· scavenging rates of SGP-1 and S-SGP were 43.79% and 33.74%, respectively. With the increase in polysaccharide concentration, the activity of the polysaccharide also gradually increased. Specifically, at a concentration of 4 mg/mL, the DPPH· scavenging rate of S-SGP (75.24%) was obviously higher than that of SGP-1 (59.53%). When the concentration of polysaccharide was 2 mg/mL, the clearance rate of ·OH showed a certain upward trend. At this time, the ·OH clearance rates of SGP-1 and S-SGP were 25.32% and 15.27%, respectively; when the polysaccharide concentration reached 4 mg/mL, the ·OH clearance rates of SGP-1 and S-SGP increased to 29.68% and 17.25%, respectively. When the polysaccharide concentration was 2 mg/mL, the clearance rate of O_2_^−^· increased slightly. At this time, the O_2_^−^· clearance rates of SGP-1 and S-SGP· were 30.79% and 32.67%, respectively. When the polysaccharide concentration was 4 mg/mL, the O_2_^−^· clearance rate of S-SGP (41.52%) was higher than that of SGP-1 (35.34%). Briefly, the scavenging abilities of SGP-1 and S-SGP against DPPH radicals, ·OH and O_2_^−^· were enhanced with increasing polysaccharide concentration. With the introduction of sulfate groups, the DPPH scavenging activity and O_2_^−^· scavenging activity of S-SGP were enhanced. Similar observations have been reported in some sulfated mushroom polysaccharides [54]. However, the hydroxyl radical scavenging activity did not show an increase, which may be attributed to the structural changes of the polysaccharide caused by the acidic conditions during sulfation. These structural changes could potentially impact its activity.

In summary, our findings indicate that the introduction of sulfate groups into polysaccharides leads to a decrease in the molecular weight of SGP-1, likely due to the acidic conditions during sulfation. Furthermore, the modified polysaccharides exhibit enhanced antioxidant activity. Consistent with our findings, the sulfated polysaccharides derived from the edible mushroom *Pleurotus ostreatus* showed a similar pattern, with a decrease in molecular weight and an increase in DPPH scavenging activity and O_2_^−^· scavenging activity, while the ·OH scavenging activity did not show significant changes [55]. Additionally, sulfation modification of *S. rugosoannulata* polysaccharides also resulted in improved antioxidant activity [22]. Therefore, sulfation modification of polysaccharides represents a promising approach for enhancing their antioxidant activity.

### 3.4. Growth Inhibition of HepG2 Cells, MDA-MB-231 Cells and A549 Cells by S-SGP

**Figure 4:**
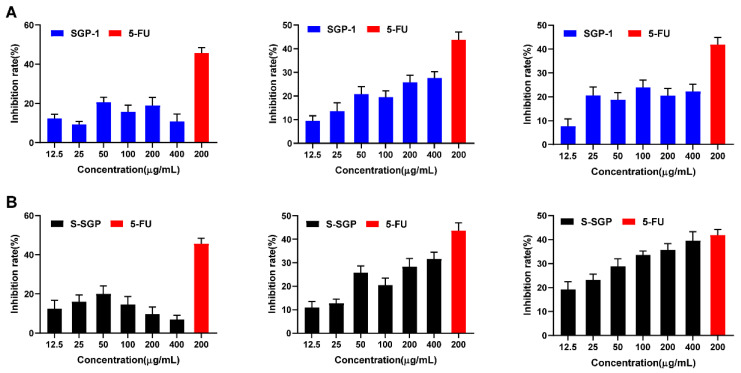
SGP and S-SGP inhibit the activity of HepG2, MDA-MB-231 and A549 cells. (**A**) In vitro growth inhibitory effects of SGP-1 on HepG2, MDA-MB-231 and A549 cells, respectively; (**B**) in vitro growth inhibitory effects of S-SGP on HepG2, MDA-MB-231 and A549 cells, respectively.

The CCK-8 method was used to measure the in vitro growth inhibitory effects of polysaccharides before and after sulfate modification on three cancer cells. Different concentrations of SGP-1 and S-SGP (12.5, 25, 50, 100, 200 and 400 μg/mL) were co-cultured with HepG2 cells, A549 cells and MDA-MB-231 cells for 48 h, respectively. The CCK-8 method detected the effect of polysaccharides in cell proliferation, as shown in Figure 4, with the concentration of polysaccharide and positive control 5-FU as the abscissa, and the inhibition rate (%) on cell proliferation as the ordinate.

Figure 4A shows the in vitro growth inhibitory effect of SGP-1 on HepG2, MDA-MB-231 and A549 cells, respectively; Figure 4B shows the in vitro growth inhibitory effect of S-SGP on HepG2, MDA-MB-231 and A549 cells, respectively. The figure reveals that SGP-1 and S-SGP at various concentrations exhibit a certain degree of inhibition on HepG2 cell proliferation. However, the inhibitory effect is not significant, and no significant correlation is observed between the dose and the effect. Therefore, no further comparison is made. Each concentration of SGP-1 and S-SGP inhibited the proliferation of MDA-MB-231 cells, and with the increase in polysaccharide concentration, the inhibition rate increased in a dose-dependent manner; when S-SGP was 400 μg/mL, the inhibition rate reached 31.62%, and the IC_50_ was 747.48 μg/mL. Different concentrations of SGP-1 and S-SGP have inhibitory effects on A549 cells proliferation, and S-SGP has a strong inhibitory effect with a significant dose-effect relationship; when S-SGP is 648 μg/mL, the inhibition rate reaches 39.5%, IC_50_. It was 577.28 μg/mL, and the anti-tumor effect was significant. Therefore, A549 cells were selected to study the anti-tumor effect of polysaccharides before and after sulfate modification.

### 3.5. S-SGP Induces Apoptosis in A549 Cells

**Figure 5:**
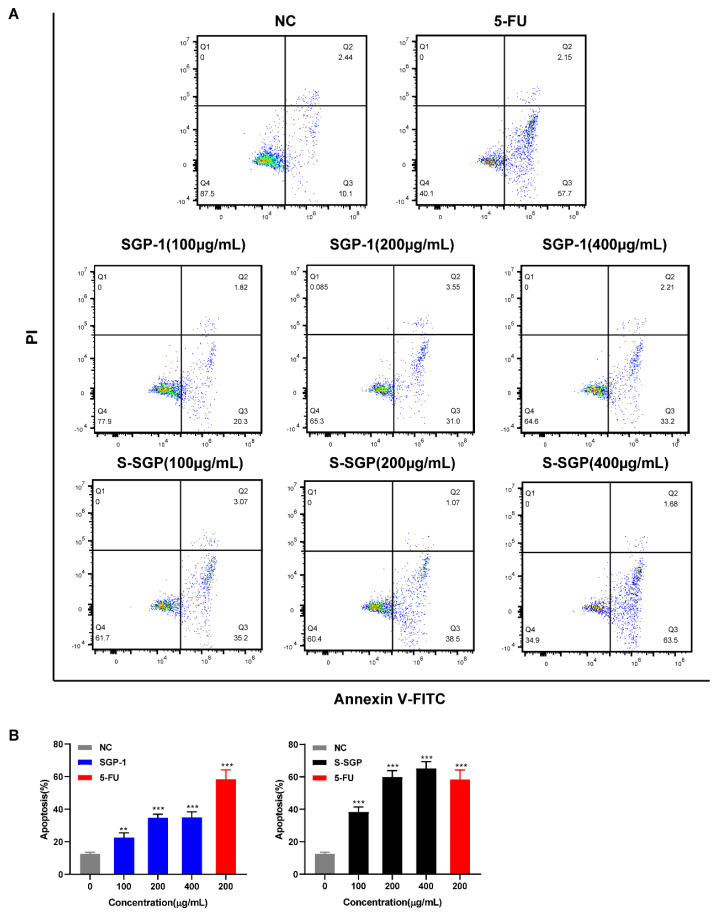
SGP-1 and S-SGP induce apoptosis in A549 cells. (**A**) Flow cytometry detected cell apoptosis in each group; (**B**) the effect of SGP-1 and S-SGP on apoptosis of A549 cells. Compared with the negative control (NC) group, ** *p* < 0.01, *** *p* < 0.001.

Apoptosis is an important physiological process and is considered the preferred method of eliminating cancer cells. Figure 5 and Table 2 show the effects of SGP-1 and S-SGP on the apoptosis of A549 cells. 

As shown in Figure 5A, after 400 μg/mL S-SGP was co-cultured with A549 cells for 48 h, the measured apoptosis rate of A549 cells reached 65.18%, which was obviously higher than that in the negative group and the SGP-1 treatment group at the same concentration. It shows that S-SGP can induce apoptosis of A549 cells and exert anti-tumor activity, and the dose is positively correlated.

**Figure 6:**
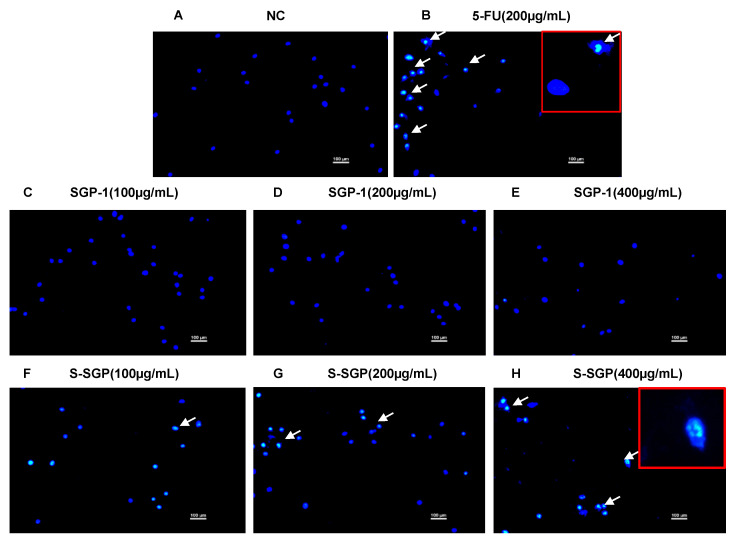
The nuclear morphology of A549 cells in each group was observed by DAPI staining. (**A**) NC group. (**B**) 5-FU group (200 μg/mL). (**C**–**E**) SGP-1 group (100 μg/mL, 200 μg/mL, and 400 μg/mL). (**F–H**) S-SGP group (100 μg/mL, 200 μg/mL, and 400 μg/mL) (DAPI-stained nuclei show blue fluorescence; typical apoptotic cells are indicated by white arrows).

The effects of SGP-1 and S-SGP on the apoptosis of A549 cells were observed by DAPI staining. As shown in Figure 6A, the nuclei of the negative control group (NC) were uniformly stained and had normal morphology. Figure 6C–E showed that the nuclei of different concentrations of SGP-1 treatment groups also showed the same state; as shown in Figure 6B, the nuclei of the 5-FU group were solidified, fragmented, hemispherical and crescent-shaped nuclei, which were typical apoptotic nuclei; as shown in Figure 6F–H, the nuclei of each dose of S-SGP showed different degrees of nuclei pyknosis, with increasing dose, more apoptotic cells.

**Figure 7:**
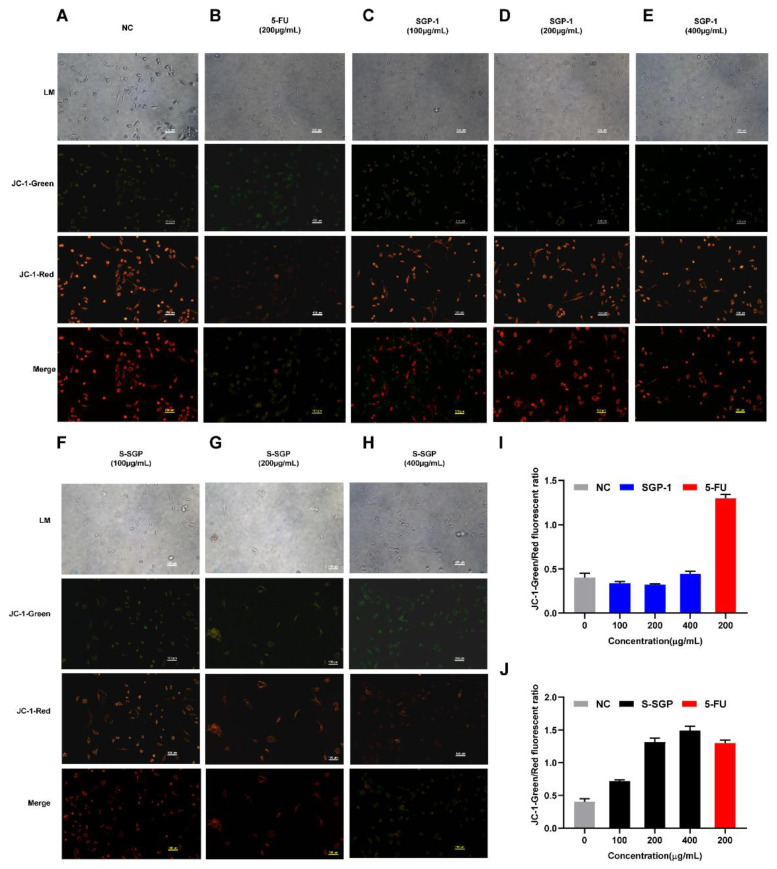
The mitochondrial membrane potential (ΔΨm) of A549 cells was detected by fluorescence inverted microscope. (**A**) NC group. (**B**) 5-FU group (200 μg/mL). (**C**–**E**) SGP-1 group (100 μg/mL, 200 μg/mL, and 400 μg/mL). (**F**–**H**) S-SGP group (100 μg/mL, 200 μg/mL, and 400 μg/mL). (**I**) JC-1-Green/ Red fluorescent ratio of NC, SGP-1, and 5-FU group. (**J**) JC-1-Green/ Red fluorescent ratio of NC, S-SGP, and 5-FU group. Note: negative control group (NC); positive control group (5-FU); sulfated *Siraitia grosvenorii* polysaccharide group (S-SGP); *Siraitia grosvenorii* polysaccharide group (SGP-1). The first row from top to bottom is the light microscope image (LM); the second row is the green fluorescence image; the third row is the red fluorescence image; the fourth row is the red-green fluorescence combined image (Merge).

The mitochondrial membrane potential of A549 cells was assessed using JC-1 staining, as depicted in Figure 7. Compared with the NC group (Figure 7A), the red fluorescence of SGP-1 in each dose group (Figure 7C–E) had no significant change to green fluorescence, when A549 cells were treated with 5-FU (Figure 7B) and each dose group of S-SGP (Figure 7F–H), the green fluorescence was enhanced and the red fluorescence was weakened. As demonstrated in Figure 7J, when the S-SGP concentration was 400 µg/mL, the JC-1 fluorescence green/red ratio increased significantly, suggesting S-SGP has a notable impact on reducing the mitochondrial membrane potential in A549 cells, leading to the induction of apoptosis.

**Figure 8:**
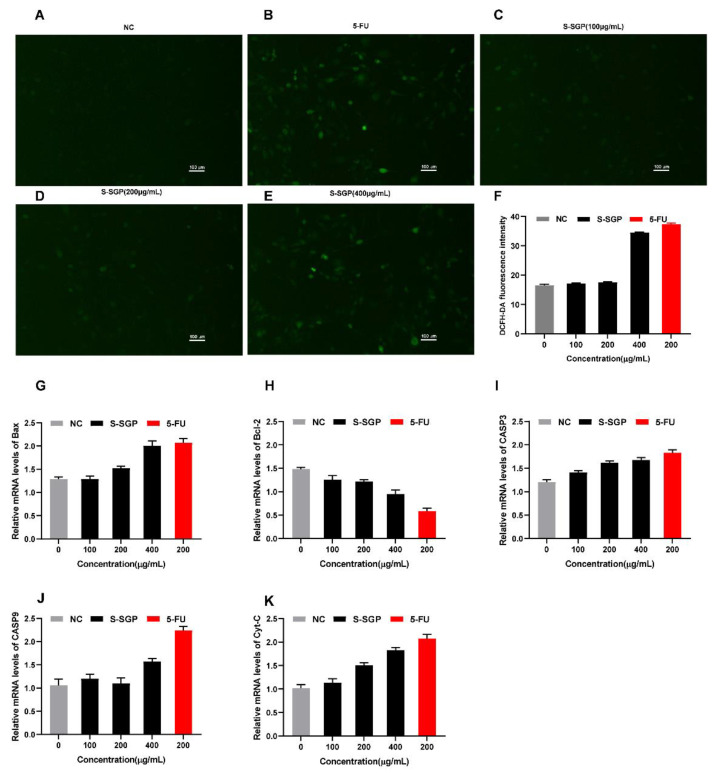
Reactive oxygen content and apoptosis-related gene expression in A549 cancer cell lines after S-SGP treatment. (**A**–**E**) The content of ROS in A549 cells from each group was assessed using DCFH-DA staining; (**F**) the effect of S-SGP on the content of reactive oxygen species in A549 cells; (**G**–**K**) relative mRNA expression levels of apoptosis-related genes in A549 cancer cell line treated by S-SGP.

Apoptosis is closely associated with the redox state of the intracellular environment. The results of the ROS experiment are presented in Figure 8A–E. When observed under a 100x microscope, the NC group exhibited a low number of fluorescent cells with weak intensity. After the S-SGP treatment, there appeared more green fluorescent cells, as shown in Figure 8F, showing a gradual increase in the fluorescence intensity as the polysaccharide concentration increased and that S-SGP significantly increased the ROS content in A549 cells.

Apoptosis is a tightly regulated process that occurs naturally to maintain internal homeostasis. It involves the activation, expression, and regulation of a cascade of genes. The mitochondrial pathway is one of the well-established pathways, and two protein families, Bcl-2 and caspases, are key factors at play [56]. To explore the underlying mechanism of S-SGP-induced apoptosis in lung cancer cells, A549 cells were employed as a model and conducted qRTPCR to assess the transcription levels of several genes involved in the apoptosis pathway. The results are presented in Figure 8G–K. The results show: Compared with controls, Bax, Cyt-C, CASP9 and CASP3 mRNA levels were significantly increased in positive control and S-SGP-treated cells, while RNA transcript levels of the Bcl-2 gene were decreased. When S-SGP was 400 μg/mL, the gene expression levels of Bax, Cyt-C, CASP9 and CASP3 were 1.65 times, 1.76 times, 1.46 times and 1.39 times that of the NC group, respectively. While the gene expression levels of Bcl-2 are 0.67 times that of the NC group. It was shown that S-SGP could change the expression levels of the above genes and induce apoptosis compared with untreated cells.

**Figure 9:**
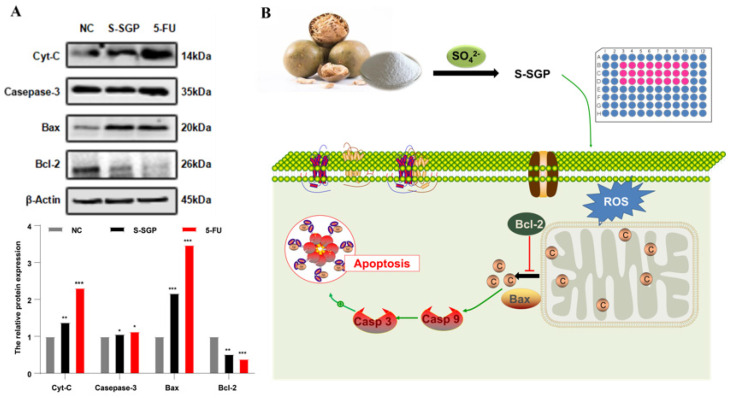
Mechanism of action of S-SGP in inducing apoptosis in A549 cells. Upon receiving apoptotic signals (such as ROS) stimulation, cells initiate the mitochondrial apoptotic pathway. S-SGP facilitates the relocalization of Bax to the surface of mitochondria, where it forms pores in the mitochondrial outer membrane. This event leads to a reduction of membrane potential and an increase in membrane permeability, resulting in the release of cytochrome C from mitochondria. Consequently, cytochrome C activates Casp-9, which subsequently activates Casp-3, ultimately culminating in cell apoptosis and exerting an anti-tumor effect. Bax: Bcl-2-associated protein X; ROS: reactive oxygen species; Bcl-2: B-cell lymphoma 2; C: cytochrome c; Casp: caspase.

As shown in Figure 9A, the bands obtained by Western blot were measured by the gel image processing system. The ratio of the gray value of the protein band to be tested and the internal reference protein *β*-actin was used as the relative expression of the protein, and the ratio of the NC group was set to “1”, the protein expressions of Cyt-C, Caspase-3, Bax and Bcl-2 in A549 cells were calculated, and the results showed: the Bax protein expression was significantly increased after 400 μg/mL S-SGP treatment, which was 2.17 times that of the NC group, while the Bcl-2 protein expression was decreased, which was 0.52 times that of the NC group. Compared with the NC group, S-SGP exhibited a significant increase in the Bax/Bcl-2 ratio, thereby promoting cell apoptosis. The protein expressions of Caspase-3 and Cyt-C were further detected, and the results showed that the protein expressions of Caspase-3 and Cyt-C were increased compared with the NC group.

Figure 9B is a schematic diagram of S-SGP-induced apoptosis of A549 cells. The cell receives apoptosis-related signal stimulation (ROS, etc.) and triggers the apoptosis signal transduction pathway (mitochondrial apoptosis pathway), resulting in changes in mitochondrial membrane permeability, the membrane potential decreases, and the expression of Bax/Bcl-2 increases, so that the apoptosis-related protein (cytochrome C) which exists in mitochondrial is released from the mitochondria to the cytoplasm, and then activates the Caspase family initiator (Caspase family, Caspase-9, etc.), further activate downstream effector enzymes (Caspase-3, etc.), and finally activate the Caspase cascade, which irreversibly causes apoptosis.

It is evident that SGP-1, after sulfation modification, exhibits enhanced anti-tumor activity. Mushrooms are known to contain abundant polysaccharides and have been a focus of polysaccharide research. Studies have revealed that polysaccharides from *Pleurotus* mushrooms also possess significant anti-tumor activity, which may be attributed to their immunomodulatory effects [57,58]. In addition to sulfation modification, polysaccharides can undergo various structural modifications such as acetylation and methylation. Polysaccharides extracted from *Calocybe indica*, after methylation modification, demonstrate notable anti-tumor effects [59]. Furthermore, Ganoderma lucidum polysaccharides, following acetylation and carboxymethylation modifications, show enhanced antioxidant and anti-tumor activities [60].

## 4. Conclusions

In this experiment, a neutral polysaccharide, SGP-1, was extracted from *Siraitia grosvenorii*. SGP-1 has a molecular weight of 3.30 × 10^4^ Da. The structure of SGP-1 is likely a glucan with 4-, 6- and 4,6-linked glucose units. The sulfate derivatives of SGP-1 were prepared by the chlorosulfonic acid method. FT-IR showed that sulfation did occur. The DS of the sulfated derivative was 0.62 and the Mw was 1.34 × 10^4^ Da. Antioxidant activity studies have shown that S-SGP has more antioxidant capacity than SGP-1. The in vitro experiments S-SGP also showed a significant inhibitory effect on A549 cells. Annexin V-FITC/PI staining and JC-1 staining experiments showed that A549 cells were treated with S-SGP to induce apoptosis. These results indicated that S-SGP reduced the mitochondrial membrane potential of A549 cells, induced apoptosis, and thus enhanced their anti-tumor activity. Therefore, the sulfated derivatives of SGP-1 are anticipated to have potential as adjuvants in the further treatment of cancer, offering valuable insights for the comprehensive exploration and utilization of *Siraitia grosvenorii*.

## Figures and Tables

**Table 1:** Analysis of the results of polysaccharide linkage sites.

RT	Methylated Sugars	Mass Fragments (*m*/*z*)	Mole Ratio	LINKAGES
24.396	1,5-Di-O-acetyl-1-deuterio-2,3,4,6-tetra-O-methyl-D-glucitol	43, 71, 87, 101, 117, 129, 145, 161, 205	0.486	Glc*p*-(1→
30.305	1,4,5-Tri-O-acetyl-1-deuterio-2,3,6-tri-O-methyl-D-glucitol	43, 87, 99, 101, 113, 117, 129, 131, 161, 173, 233	0.359	→4)-Glc*p*-(1→
31.340	1,5,6-Tri-O-acetyl-1-deuterio-2,3,4-tri-O-methyl-D-glucitol	43, 87, 99, 101, 117, 129, 161, 189, 233	0.060	→6-Glc*p*-(1→
37.506	1,4,5,6-Tetra-O-acetyl-1-deuterio-2,3-di-O-methyl-D-glucitol	43, 71, 85, 87, 99, 101, 117, 127, 159, 161, 201	0.095	→4,6)-Glc*p*-(1→

**Table 2:** Effects of S-SGP on the apoptosis rates of A549 cells (x ± s, *n* = 3).

Group	Early Apoptosis Rate (%)	Late Apoptosis Rate (%)	Total Apoptosis Rate (%)
NC	10.1 ± 1.75	2.44 ± 1.83	12.54 ± 0.98
5-FU (200 µg/mL)	57.5 ± 3.64	2.15 ± 1.56	58.36 ± 5.83 ***
SGP-1 (100 µg/mL)	20.3 ± 2.67	1.82 ± 1.08	22.12 ± 2.51 **
SGP-1 (200 µg/mL)	31.0 ± 3.05	3.55 ± 1.66	34.55 ± 2.67 ***
SGP-1 (400 µg/mL)	33.2 ± 3.19	2.21 ± 1.73	35.41 ± 3.13 ***
S-SGP (100 µg/mL)	35.2 ± 4.16	3.07 ± 1.71	38.27 ± 3.11 ***
S-SGP (200 µg/mL)	38.5 ± 3.26	1.07 ± 1.05	59.85 ± 3.98 ***
S-SGP (400 µg/mL)	63.5 ± 4.25	1.68 ± 1.50	65.18 ± 4.12 ***

Note: data were recorded as mean values ± standard deviations. In the column of “total apoptosis rate (%)”, compared with the negative control (NC) group, ** *p* < 0.01, *** *p* < 0.001.

## Data Availability

Data is contained within the article or Supplementary Materials.

## Supplementary Materials

The following supporting information can be downloaded at: https://www.mdpi.com/article/10.3390/foods12112133/s1, Table S1: Primer sequence.
