# Supplementary material for: Structure Characterization, In Vitro Antioxidant and Anti-Tumor Activity of Sulfated Polysaccharide from Siraitia grosvenorii
Source: Foods. 2023 May 25;12(11):2133. doi: 10.3390/foods12112133 (PMC10252265; doi:10.3390/foods12112133)
Supplement: Supplementary file 1 [file foods-12-02133-s001.zip › foods-2376365-supplementary.pdf]

Supplementary Materials

Table S1. Primer sequence.

Gene	Primer sequences (5′ to 3′)
β-Actin-F	CATGTACGTTGCTATCCAGGC
β-Actin-R	CTCCTTAATGTCACGCACGAT
Bax-F	CCCGAGAGGTCTTTTTCCGAG
Bax-R	CCAGCCCATGATGGTTCTGAT
Bcl-2-F	GGTGGGGTCATGTGTGTGG
Bcl-2-R	CGGTTCAGGTACTCAGTCATCC
CASP3-F	TTCAGAGGGGATCGTTGTAGAAGTC
CASP3-R	CAAGCTTGTCGGCATACTGTTTCAG
CASP9-F	CACCCAGACCAGTGGACATT
CASP9-R	TGCTCAGGATGTAAGCCAAATCT
Cyt-C-F	AACTGGCCTCTGACAGGCAAT
Cyt-C-R	TCAGTGGTGAAAGGCAGCATC
